# Predicting the protective behavioral intentions for parents with young children that possess different levels of education in Hong Kong using the theory of planned behavior for air polluted with PM_2.5_

**DOI:** 10.1186/s12889-022-13141-9

**Published:** 2022-04-15

**Authors:** Siu-Kei Woo, Ben LePage, Yi-Te Chiang, Wei-Ta Fang

**Affiliations:** 1 Graduate Institute of Environmental Education, National Taiwan Normal University, Taipei, 116 Taiwan; 2 Academy of Natural Sciences, 1900 Benjamin Franklin Parkway, Philadelphia, PA 19103 USA

**Keywords:** PM_2.5_ behavioral intentions, Undergraduate and graduate environmental education, Theory of Planned Behavior, Young children’s parents

## Abstract

**Background:**

Air pollution has fast become an issue with great environmental and human health problems that can be attributed to rapid global industrialization and urbanization that has strong negative impacts on human health. Children are particularly vulnerable. While studies on the effects and toxicology of particulate matter pollutants that are 2.5 microns or smaller in size (PM_2.5_) are abundant, understanding the factors that influence human behaviors against or the avoidance of exposure/contact to air polluted with high levels of PM_2.5_ is lacking. In this study, this gap was narrowed by used the Theory of Planned Behavior (TPB) to investigate the effects of Attitudes (AT), Subjective Norms (SN), and Perceived Behavioral Controls (PBC) on the Behavioral Intentions (BI) of parents with young children with different levels of education against or avoiding contact/exposure to air polluted with high levels of PM_2.5_.

**Methods:**

The TPB model was used to predict the BI of parents with young children with different levels of education that live in Hong Kong using the results collected from 410 online questionnaires. Aspects of the BI influencing parents with young children that earned undergraduate group and post-graduate group were analysed using Smart Partial Least Squares 2.0 software.

**Results:**

Our results revealed there were substantial differences in the AT of parents with young children that earned undergraduate and post-graduate degrees with respect to exposure to air with PM_2.5_ pollution.

**Conclusions:**

In this study we assessed the factors that influence the air pollution prevention and avoidance behaviors of parents of the undergraduate and post-graduate groups that were exposed to air polluted with PM_2.5_. Our results show the AT, SN, PBC, and BI used in the air pollution protection model for the parents of both groups are connected by separate pathways. The undergraduate group has a higher PBC compared to the post-graduate group because the SN associated with their social ecosystems contribute to their BI. Using path analysis, we revealed that the undergraduate and post-graduate groups had different BI paths. The BI path of the undergraduate group is purer and simpler when compared with the path of the post-graduate group.

**Supplementary Information:**

The online version contains supplementary material available at 10.1186/s12889-022-13141-9.

## Background

Ajzen [1] created the TPB in 1991 from the Rational Choice Theory. To predict BI, the TPB is a commonly used research model [1, 2]. Fang [2] pointed out that TPB is more passive than Hines et al.’s [2] Responsible Behavior Model. However, according to Fang [2] TPB has a rigorous calculation method and has an advantage in quantitative research on predicting behavior. Therefore, this theory is suitable for marketing and health promoting policies that require accurate data [2]. The TPB has since been widely applied in the research on health management behaviors such as sex [3, 4], smoking [5, 6], alcoholism[7], and drug abuse [8]. In addition, anti-PM_2.5_ behavioral intentions comprise not only health protection behaviors, but also the environmental-friendly behaviors of individuals [9]. TPB is currently one of the most commonly used theories in research studies on the interpretation and prediction of various environmental-friendly behaviors [10, 11].

As such air pollution has fast become a substantial environmental problem that can be attributed to rapid global industrialization and urbanization and has a strong negative impact on the environment and human health, particularly children [12, 13]. Contaminants such as volatile and semi-volatile organic compounds, dust, and particulate matter have seriously impacted the environment and human health. Among these, PM_2.5_ are concern because with each breath that we take, the fine particles that pollute the air enter deep the into our lungs. PM_2.5_ are common byproduct of car exhaust and the incomplete combustion of wood, but dust, pollen, and spores also fall into this group. The air polluted with PM _2.5_ that we sometimes see in the air is largely composed of PM_2.5_ pollution. Inhalation of air pollution with PM_2.5_ are associated with respiratory problems, premature mortality, increased hospital admissions for heart or lung disease, acute and chronic bronchitis, and asthma [14]. As such, reducing PM_2.5_ pollution should bring huge economic benefits to society and avoid/reduce human mortality [15].

The World Health Organization [16] reported that there were 3.1 million deaths globally that could be attributed to air polluted with PM_2.5_ and in the EU alone, 307,000 premature deaths resulted from air polluted with PM_2.5_ in 2019 [17]. Although PM_2.5_ are global issue, in 2020 the 50 most polluted cities in the world based on PM_2.5_ levels included 35 cities are from India, 7 from China, 5 from Pakistan, and 2 from Bangladesh and 1 from Indonesia [18]. Zhang et al.’s [19] analyses of the 33 megacities (populations > than 10 million people) based on long-term remote sensed observations indicated that the PM_2.5_ concentrations did not improve between 1998 to 2018 and 452 million (M) people were exposed to PM_2.5_ concentrations > 10 µg.m^3^, which is the WHO’s upper limit for a healthy atmosphere and 162 M people were exposed to concentrations > 35 µg/m^3^, which is the WHO’s non-attainment air quality threshold. Although there has been considerable work on the PM_2.5_ levels in megacities and regions of the world where air pollution is a big problem, PM_2.5_ can impact the air quality anywhere in the world and we’d be remiss to preclude the human health impacts of PM_2.5_ air pollution from anywhere in the world.

The reduction of PM_2.5_ contributions and protection against air polluted with PM_2.5_ has become the focus for governments and the public at large [20]. Environmental problems in Hong Kong are mostly focused on the management and less on understanding environmental behaviors. In other words, the reactions and responses of citizens and their behaviors on environmental issues rather than their literacy or understanding of environmental issues. Therefore, in this study we focused on understanding how citizens responded to air pollution concerns, especially how parents with young children and with different levels of education responded or reacted when their children were exposed to air polluted with PM_2.5_. Children are one of the most sensitive receptors and short-term exposure to air polluted with PM_2.5_ can trigger asthma, requiring hospitalization [21], and younger children are more likely to be affected than older children [22].

Although studies on behaviors reducing or avoiding with PM_2.5_ have been explored from the perspective of the family [9], studies on the behavioral and health management elements related to protection against or avoiding air polluted with PM_2.5_ are lacking. We believe that the pathways that influence human behaviors that avoid or minimize exposure to PM_2.5_ is an ideal topic to study in more detail. In addition to reducing and controlling exposure to air contaminated with PM_2.5_, it was also important to understand the elements that generated the protective behaviors to avoid exposure to air polluted with PM_2.5_. As such we focused on parents with young children with different levels of education that were living in Hong Kong. We also considered the parent’s health concerns and the hazards associated with exposure to air polluted with PM_2.5_.

There is a correlation between economics and education level [23], which suggests the relationship between education and behavior is important and cannot be ignored [24]. Consequently, better understanding of the role that the level of education plays in shaping people’s behaviors towards avoiding air polluted with PM_2.5_ was considered important because this type of analysis has not yet been performed. Furthermore, education levels are related to a person’s AT, which are then affected by their SN [25]. Therefore, we hypothesised that the parent’s level of education affected their SN positively. To understand parental behaviors related to avoiding air polluted with PM_2.5_, we assessed the protective behaviors of parents with young children with different levels of education living in Hong Kong. The dimensions that shape protective behaviors included: (1) attitudes (AT), (2) perceived behavioral controls (PBC), and (3) subjective norms (SN). The TPB model emphasizes controlled aspects of human information processing and decision-making that is concerned with goal-oriented behaviors and behaviors that are guided by a conscious self-regulatory process. AT is the intervening variable of SN and BI because a person’s AT can affect their BI directly or indirectly through their SN [26–31].

**Fig. 1:**
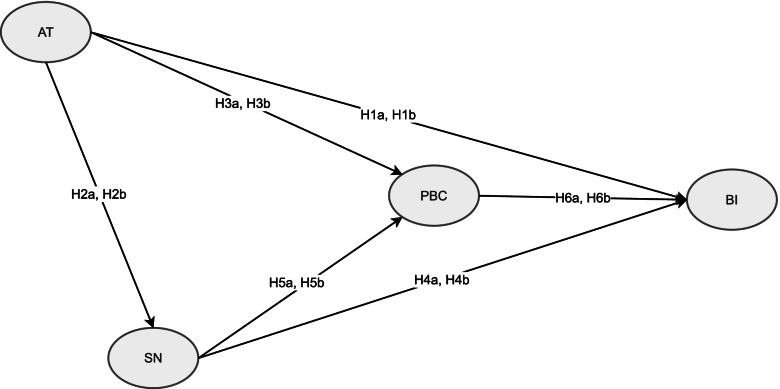
Study Hypotheses. (AT: attitudes, SN: subjective norms, PBC: perceived behavioral controls, BI: behavioral intentions)

The use of this model may help explain the relationship between social psychology and people’s preventative behaviors related to environmental problems. Despite the plethora of studies assessing the toxicity and impact of air polluted with PM_2.5_ on humans and the environment, work on the factors that influence people’s behaviors such as AT and SN towards avoiding air polluted with PM_2.5_ is lacking [12]. From the perspective of TPB, people find that even when accounting for the predictive variables in TPB, understanding past behavior(s) can help predict future behaviors [1]. SN are most weighed in predicting an individuals' willingness to reduce emissions that contribute to air pollution, but the best way to motivate people to reduce air pollution may be to exert social pressure or create a people-friendly atmosphere and environmental protection activities [32]. The use of the TPB model with Hong Kong’s societal norms and values indicate subjective regulations may be the most important contribution for people to reduce air pollution emissions. Therefore, we tested the following hypotheses (Fig. 1).

### Hypothesis 1a, and H1b (H1a, H1b)

The undergraduate group (H1a) and post-graduate group (H1b) possess AT that can affect their respective BI [26–31]. There are considerable data that demonstrate SN and AT are significantly related [33–35] and AT affects their SN positively [34, 36].

### Hypothesis 2a, 2b (H2a, H2b)

The undergraduate group (H1a) and post-graduate group (H1b) possess AT can affect their SN. The results of several studies show that people’s SN and AT are significant and positively related [33–36].

### Hypothesis 3a, 3b (H3a, H3b)

AT can affect the PBC of the undergraduate group (H1a) and post-graduate group (H1b). The results of studies indicate people’s SN and PBC are significantly related [33–35] and affect their PBC directly and BI directly and indirectly [9, 37, 38].

### Hypothesis 4a, 4b (H4a, H4b)

The undergraduate group (H1a) and post-graduate group (H1b) possess SN that can affect their respective BI. Their SN and PBC affect BI [28, 39], but it is not yet clear whether their PBC affects their SN positively in the study. Nonetheless, we hypothesised that the PBC of parents with young children and with different levels of education living in Hong Kong affect their SN positively. The results of studies have shown that people’s SN and PBC are significantly related [33–35].

### Hypothesis 5a, 5b (H5a, H5b)

The undergraduate group (H1a) and post-graduate group (H1b) follow SN that can affect their respective PBC. Their SN affects their PBC directly and affect BI indirectly [37].

### Hypothesis 6a, 6b (H6a, H6b)

The undergraduate group (H1a) and post-graduate group (H1b) possess PBC that can affect their respective BI. The PBC of parents of both groups affects their BI directly [9, 37, 38].

The TPB model was used to identify key factors such as AT, SN, and PBC that influence the behavior of parents of both groups to protect their children against air pollution, especially air polluted with PM_2.5_.

### Methodology

The questionnaires were collected using a purposive, stratified and snowball methodology [40]. The development, delivery, and collection of the questionnaires follow Liu’s 2018 approach [14]. We contacted the parents of both groups via Facebook and WhatsApp to ask whether they would be interested and willing to participate in our survey. Those that agreed were then sent an electronic invitation card using the Facebook and WhatsApp, which are the social media platforms that are the most used by Hong Kong residents. We received 410 responses and the parents of these groups agreed to participate in our survey and completed the questionnaires online.

Although this research did not involve invasive human studies, we obtained parental consent to participate in this research using an anonymous answering method that was submitted online. The National Taiwan Normal University Research Ethics Committee deemed that this work was not within the scope of the "Human Research Law" and the committee then approved the research protocols and agreed to provide informed consent to the parents and their right to opt out of the study (No. 201804HS008).

The questions in the questionnaire were adapted from previous environmental behavior surveys, especially those involving AT [41], SN [42], and environmental behavior [41]. The questionnaire was reviewed by six experts in health promotion, sanitation education, and environmental education to determine the validity of the questions posed. Following this peer review process the questionnaire underwent subtle changes that were focused on simplifying the wording and terms that were being used locally prior to distributing the questionnaire. These changes ultimately helped the parents of both groups participating in the survey better understand the questions that were being asked while retaining the context and/or original meaning of the questions. After the questionnaire was reviewed by the experts and revised, a pilot test was suggested by the experts, to determine whether there was uncertainty in the questions being asked.

We used Statistical Package for the Social Sciences (SPSS) 23 for the statistical analysis and descriptive statistics were used to calculate the number of occurrences, percent, average, and standard deviation (SD) scores of the questionnaire responses. A single-factor variance analysis was used to determine differences in AT, SN, PBC, and BI of the parents with young children and with different levels of education, living in Hong Kong. The Pearson Correlation Coefficient was then used to measure the existence of these key dimensions, their strength, and direction of the relationships. We choose education as a key factor because it was an underlying variable that played a role in many of the results. This will be discussed later.

A five-point Likert scale (1 = strongly disagree to 5 = strongly agree) was used as our measurement tool. We then used SmartPLS2.0 statistical software for path and statistical analyses to predict the influence of AT, SN, and PBC on BI. PLS-SEM (Partial least squares regression) is an exploratory multivariate method that can build SEM in research. This type of research is critically important as the research questions posed in research involving small populations is often about serious health concerns in vulnerable and underrepresented populations [43].

## Results

We received 301 responses from the undergraduate group and 109 responses from the post-graduate group. The Cronbach α of the AT (Table 1), SN (Table 2), PBC (Table 3), and BI (Table 4) variables for questions posed to the parents of both groups support the reliability of the questions and responses of questionnaire responses [43]. Cronbach α is a measure of the internal consistency or reliability of questions that are based on a Likert scale. The Cronbach α value of the questionnaire was 0.928, which exceeds the 0.7. Values above 0.7 are considered good.

**Table 1:** Results of Attitudes (AT) Questions

Attitudes	Undergraduate (*n* = 301)		Post-graduate (*n* = 109)	
	**Mean**	**SD**	**Mean**	**SD**
AT 1. I am worried about air pollution in Hong Kong	4.29	0.78	4.33	0.64
AT 2. I care about recycling and utilization of daily life resources	4.17	0.87	4.24	0.73
AT 3. I care about whether the air polluted with PM _2.5_ affects the health of myself and my family	4.42	0.78	4.35	0.71
AT 4. I am concerned about whether the air polluted with PM _2.5_ affects the health of community residents	4.27	0.81	4.17	0.74
AT 5. I care about whether the air polluted with PM _2.5_ affects the health of the citizens	4.30	0.76	4.22	0.71
AT 6. I care about environmental issues arising from industry and manufacturing	4.29	0.78	4.22	0.79
AT 7. I care about environmental issues arising from economic development	4.21	0.77	4.27	0.75
AT Scores	4.28	0.65	4.26	0.58

**Table 2:** Results of Subjective Norms (SN) Questions

Subjective Norms	Undergraduate (*n* = 301)		Post-graduate (*n* = 109)	
	**Mean**	**SD**	**Mean**	**SD**
SN 1. Most people who are important to me support me by not eating barbecued food	3.58	0.92	3.46	0.88
SN 2. Most people who are important to me support me by walking, cycling or taking public transportation to go out	3.88	0.89	3.92	0.88
SN 3. Most people who are important to me support me when I wear masks for myself and my children when air pollution occurs	4.14	0.84	3.91	0.95
SN 4. Most people who are important to me support me when I participate in environmental protection activities to improve air pollution	3.86	0.88	3.80	0.95
SN Scores	3.86	0.73	3.77	0.79

**Table 3:** Results of Perceived Behavioral Controls (PBC) Questions

Perceived Behavioral Controls	**Undergraduate (*n* = 301)**		**Post-graduate (*n* = 109)**	
	**Mean**	**SD**	**Mean**	**SD**
PBC 1. I can skip eating barbecued food to reduce air pollution	3.76	0.91	3.53	1.01
PBC 2. I can walk, bike, or take public transportation to reduce air pollution	4.16	0.82	4.13	0.92
PBC 3. Although wearing a mask is troublesome, I can remind my children to use a mask when the air polluted with PM _2.5_ is severe	4.31	0.85	4.05	0.92
PBC 4. When the air polluted with PM _2.5_ is severe, I can guide children to wear a mask and protect their face	4.30	0.82	4.01	0.93
PBC Scores	4.13	0.65	3.93	0.77

**Table 4:** Results of Behavioral Intentions (BI) Questions

Behavioral Intentions	Undergraduate (*n* = 301)		Post-graduate (*n* = 109)	
	**Mean**	**SD**	**Mean**	**SD**
BI 1. I don’t eat barbecued food to avoid air pollutants	3.50	0.89	3.24	0.98
BI 2. I can walk and cycle, or take public transportation to go out to reduce air pollution	3.98	0.84	3.83	0.92
BI 3. Even if I spend more, I will use fuel with less environmental impact	3.80	0.77	3.61	0.82
BI 4. I will advise others not to pollute the environment	3.98	0.79	3.83	0.76
BI 5. When the air pollution is serious, I will let the child wear a mask and protect their face	4.04	0.93	3.70	1.08
BI 6. When the Air Pollution Health Index (AQHI) reaches a "very high" level, I will let the child stay indoors (the second highest level among the five levels)	3.67	0.99	3.21	1.12
BI 7. I will pay attention to the Air Quality Health Index (AQHI) every day to remind children to pay attention to air pollution protection (AQHI)	3.49	1.07	2.82	1.23
BI Scores	3.78	0.65	3.46	0.68

### Correlation analysis

Before performing an SEM analysis, a correlation analysis of the factors is performed to check whether there is a correlation between the factors. The correlation analysis in this paper confirms that the two populations and four factors have significant positive correlations. As such, a path analysis [44], using PLS-SEM was used to verify the 12 hypotheses of this study only 2 vs 3.

A correlation analysis was performed on the average scores results of the parents from both groups. The data show that all of the factors we measured are statistically positively correlated for the parents of both groups (Tables 5 and 6). Therefore, each factor is related to the other and SEM was performed to clarify the factor paths and relationships.

**Table 5:** Parents of Undergraduate Group

	AT	SN	PBC	BI
AT	1.000			
SN	0.468***	1.000		
PBC	0.543***	0.665***	1.000	
BI	0.492***	0.662***	0.649***	1.000

^***^ = *p* < 0.001 Two-tailed test

**Table 6:** Parents of Post-graduate Group

	AT	SN	PBC	BI
AT	1.000			
SN	0.382***	1.000		
PBC	0.352***	0.721***	1.000	
BI	0.352***	0.587***	0.657***	1.000

^***^ = *p* < 0.001 Two-tailed test

### Path analysis and PLS-SEM

Aspects of the BI influencing the parents of both groups were analysed using Smart Partial Least Squares 2.0 SEM software and the results are shown in Tables 7, 8 and 9. This approach allowed us to examine the potential cause and effect relationships in path models with latent variables. The average variance (AVE) is a measure of the amount of variance that is captured by a construct in relation to the amount of variance due to the measurement error [45]. The values ​​for AT, SN, PBC, and BI of both groups are higher than 0.4, which is the acceptable value factor loadings [46], indicating that the values reached a level of convergent validity, which is the degree to which the two measures are theoretically related.

**Table 7:** PLS Analysis of the Undergraduate Group

	**AVE**	**CR**	**R**^**2**^	**Cronbach's α**
AT	0.6421	0.9248		0.6421
SN	0.7509	0.9233	0.1583	0.7509
PBC	0.6664	0.8879	0.5355	0.6664
BI	0.4611	0.8556	0.5297	0.4611

**Table 8:** PLS Analysis of the Post-graduate Group

	**AVE**	**CR**	**R**^**2**^	**Cronbach's α**
AT	0.6907	0.9393		0.6907
SN	0.6901	0.8989	0.8502	0.6901
PBC	0.5896	0.8504	0.7623	0.5896
BI	0.5229	0.8842	0.8469	0.5229

**Table 9:** Comparison of the results: the Undergraduate and Post-graduate Groups

	**AVE**	**CR**	**R**^**2**^	**Cronbach’s α**
**Undergraduate**				
AT	0.6421	0.9248		0.6421
SN	0.7509	0.9233	0.1583	0.7509
PBC	0.6664	0.8879	0.5355	0.6664
BI	0.4611	0.8556	0.5297	0.4611
**Post-graduate**				
AT	0.6907	0.9393		0.6907
SN	0.6901	0.8989	0.8502	0.6901
PBC	0.5896	0.8504	0.7623	0.5896
BI	0.5229	0.8842	0.8469	0.5229

The composite reliability (CR) is a measure of the internal consistency of a scale item and is much like Cronbach’s α [47], which is a measure of the factor variance. If each factor value is greater than 0.7, then the value indicates that the internal consistency or variation of each factor meets the credibility standard [48, 49].

The Cronbach's α of the 4 dimensions all reached a credibility standard above 0.4 [48, 49], indicating that all of the data we collected are valid. In the post-graduate group, the R^2^ of their SN was 0.8502, PBC was 0.7623, and BI was 0.8469. The R^2^ value is the proportion of the variance for a dependent variable that can be explained by an independent variable and R^2^ values above 0.75 are considered strong.

**Fig. 2:**
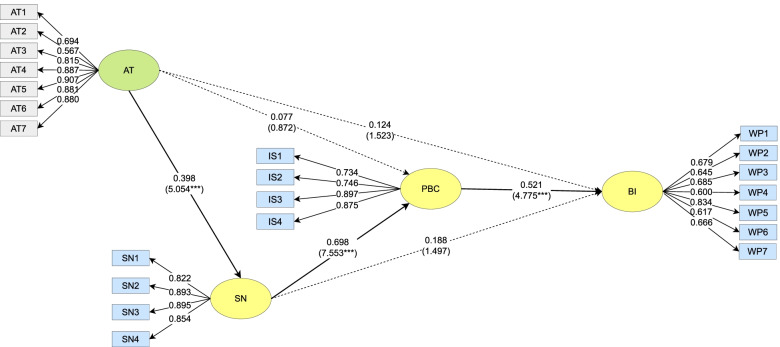
Path Coefficients of the Undergraduate Group. *** *p* < 0.001

**Fig. 3:**
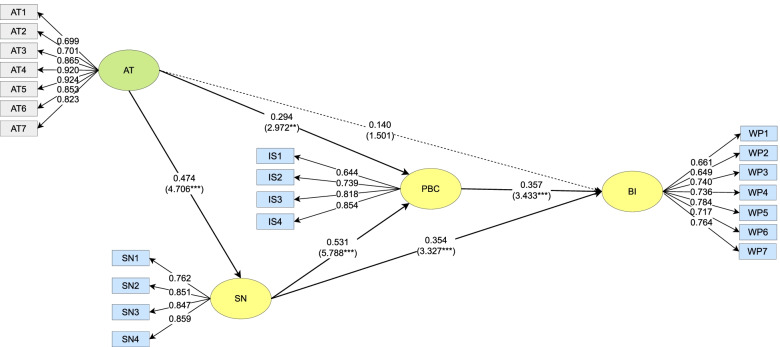
Path Coefficients of the Post-graduate Group. ** *p* < 0.01, *** *p* < 0.001

The structure of the BI models of the parents from both groups is shown in Figs. 2 and 3. The t-value of the paths were obtained using BootStrapping methodology to test the significance levels of the results. BootStrapping is a test that uses random sampling with replacements, mimicking the sampling process and falls under the broader class of resampling methods [50]). The AT of the undergraduate group had a significant positive predictive effect on their SN (*β* = 0.398, *t* = 5.054***), but no significant effect on their PBC (*β* = 0.077, *t* = 0.872) or BI (*β* = 0.124, *t* = 1.523). The SN of the undergraduate group had a significant positive predictive effect on their PBC (*β* = 0.698, *t* = 7.553***), but no significant effect on their BI (*β* = 0.188, *t* = 1.497). The PBC of the undergraduate group had a significant positive predictive effect on their BI (*β* = 0.521, *t* = 4.775***).

The AT of the post-graduate group had significant positive predictive effect on their SN (*β* = 0.474, *t* = 4.706***) and PBC (*β* = 0.294, *t* = 2.972***), but no significant effect on their BI (*β* = 0.140, *t* = 1.501). The SN of the post-graduate group had a significant positive predictive effect on their PBC (*β* = 0.531, *t* = 5.788***) and BI (*β* = 0.354, *t* = 3.327***). The PBC of the post-graduate group had a significant positive predictive effect on their BI (*β* = 0.357, *t* = 3.433***).

## Discussion

Using the TPB framework, we conducted a survey to understand how AT, SN, and PBC affect the protective behaviors of parents from both groups with respect to air polluted with PM_2.5_. The results were then mapped using a SEM to better understand the relationships and paths between AT, SN, and PBC based on the behavioral differences of the parents from both groups. Of the 12 hypotheses tested, the results supported 3 (H2a, 5a, and 6a) for undergraduate group and 5 the post-graduate group (H2b, 3b, 4b, 5b, 6b). The difference between both groups is that there are more paths between AT and AI in the post-graduate group compared to the undergraduate group.

The results indicate the SN and BI scores of the undergraduate group are statistically higher than the post-graduate group, but there were no statistically significant differences in the AT and PBC of both groups. Similar results between parents with different education levels and behavior are known too [46]. Table 9 shows the comparison of the results of parents with different levels of education.

### Influence of Attitudes (AT)

The results show there are no statistical differences in the AT of both groups with respect to protective behaviors related to air pollution in Hong Kong. The path model indicates the AT of both groups does not affect their BI directly, but their SN does. The results also indicate that the AT of both groups show impacted SN (*β* = 0.398, *t* = 5.054 > 3.29), but their PBC and BI are not directly impacted. The AT to PBC and AT to SN (*β* = 0.474, *t* = 4.706 > 3.29) paths of both groups are affected (*β* = 0.294, *t* = 2.972 > 3.29). These results mean the AT of the parents from both groups affect their BI, but both groups formed different paths; AT-SN-PBC-BI for the undergraduate group and AT-SN-PBC-BI, AT-PBC-BI and AT-SN-BI for the post-graduate group. The results also show that there is no direct path for the AT -BI path in both groups.

### Influence of Subjective Norms (SN)

The results of the independent sample T-test found that the SN of the undergraduate group are statistically higher than the post-graduate group. This suggests that the undergraduate group may have felt more social pressure from their peers to follow the SN associated with air pollution or expected behaviors compared to the post-graduate group. But there are other possibilities for this result and clarifying or better understanding this result is well outside of the scope of this study and will certainly require more attention.

In the TPB path models, the SN are directly affected by the AT [33–35] and AT affects SN positively [34, 36]. In our study the data indicate that the SN affects the PBC of both groups, but the BI of the undergraduate group is not affected.

These results show that although the average SN scores of the undergraduate group are higher than those of the post-graduate group, their SN cannot directly affect their BI and significantly affects their PBC. The SN of the post-graduate group does not directly affect their PBC, but instead they directly affect their BI. This suggests that the BI of the post-graduate group associated with air pollution are based on the expectations of people around them, while the BI of the undergraduate group may not. SN is about the expectation of important people, which means those parents’ expectations towards the opinions of people who they care the most [1].

### Influence of Perceived Behavioral Controls (PBC)

The results of this study show that there is no statistically significant difference in the PCB of air pollution prevention behaviors in both groups. The PBC for the parents of both groups affect their respective BI [9, 36, 38, 47] associated with air pollution prevention with PBC being the main factor influencing their BI [9, 37, 38].

The PBC of both groups are affected by different factors. In our analysis, the PBC of the undergraduate group was affected by their SN, which has been seen in other analyses [33–35]. This suggests a university level education may be a prerequisite to gain a PBC that influences SN, rather than AT towards air pollution protection behaviors. However, AT can positively affect SN [33, 35], which can then affect PBC. The PBC of the post-graduate group is also affected by AT and SN, which suggests that their PBC is influenced by the SN and the people around them, but that they can develop their own AT towards air pollution protection behaviors [33–35].

### Analysis of Behavioral Intentions (BI)

The results of the independent sample T-test found that the air pollution prevention BI of the undergraduate group was statistically higher than that of the post-graduate group, which means post-graduate group have better behavior than undergraduate group in towards air polluted with PM_2.5._

Path model analyses revealed two air pollution protection BI models. Only three (H2a, 5a, 6a) of the six hypotheses (H1a, 2a, 3a, 4a, 5a, 6a) in the undergraduate group model were supported and formed a single path- AT-SN-PBC-BI. The PBC in the undergraduate group was based on the SN around them, which ultimately contributes to the BI of their protective behavior.

In the post-graduate group model, the six hypotheses were supported (H1b, H2b, 3b, 4b, 5b, and 6b). Only AT to BI path was not supported (H2a). Statistically higher scores showing a more complex and diverse model. However, the BI generated in the post-graduate group shows a series of more diverse and complex pathways that have significantly lower scores than the undergraduate group, which means the decision progress of post-graduate group is more complex than those of undergraduate group. Past studies have pointed out that higher education may contribute to less understanding of issues outside of their respective professional fields. For example, business and marketing students may be less aware of the environment than a biology student [45]. Our research reveals another key factor, which is the difference between SN and BI. In our research, the SN of the undergraduate group does not directly affect their BI, but it appears that their BI is affected indirectly through their PBC [37].

This means that people in the undergraduate group probably go through a process of self-judgment to develop a BI for pollution prevention [52]. In addition to the direct influence of PBC, the BI the post-graduate group are directly affected by their SN [28, 39]. This suggests that the BI in both groups is partly derived from meeting the expectations of others around them, not just their own PBC. In other words, this is not necessarily out of their own will, but the pressure exerted by SN and cultural values.

### Limitations

According to Gifford and Nilsson [51], personal and social factors can affect pro-environmental behavior. It is worth performing more research that considers the types of factors that affect environmental behaviors toward air pollution. They also pointed out that pro-environmental behaviors may also be caused by personal non-environmental goals like improving health and saving money. While this study was focused on parental behaviors, other groups from other regions or assessment of more variables such as culture, economics, age, sex, may contribute important information about people’s behaviors towards air pollution and other environmental issues. The relationship between social norms and values, sociology, and sound science are poorly understood. As such, this research area exciting and requires researchers from many disciplines to work collaboratively to provide solutions. Nonetheless, research on any aspect in this field could take decades and still never meet the expectations of all stakeholders. Moreover, maybe there are better statistical models for comparing multi-group data.

## Conclusions

In this study we assessed the factors that influence the air pollution prevention and avoidance behaviors associated with air polluted with PM_2.5_ for parents in the undergraduate and post-graduate groups. Our results show the AT, SN, PBC and BI used in the air pollution protection model for the parents of both groups are connected by separate pathways. The undergraduate group has a higher PBC compared to the post-graduate group and because the SN associated with their social ecosystems contribute to their BI. These findings are important because we can predict the protective BI for parents with young children that possess different levels of education in Hong Kong using the TPB for air polluted with PM_2.5._

Using a path analysis tool, we revealed that the undergraduate and post-graduate groups had different BI paths. The BI path of the undergraduate group is purer and simpler when compared with the path of the post-graduate group: only three (H2a, 5a, 6a) of the six hypotheses (H1a, 2a, 3a, 4a, 5a, 6a) in the undergraduate group model forming a single path AT-SN-PBC-BI. The PBC in the undergraduate group form based on the SN around them, which ultimately contributes to their BI to the protective behavior. Meanwhile, in the post-graduate group model, the six hypotheses including H2b, 3b, 4b, 5b, 6b are statistically higher scores, which forming paths including AT-SN-PBC-BI, AT-PBC-BI and AT-SN-BI. But there is no path for AT-BI (H1a, 1b) in both groups.

In this study, AT which ultimately contributes to their SN in both groups more than contributes to the PBC and BI in the study. These findings are important because AT cannot affect BI that means attitude itself cannot affect protective behavior associated with PM_2.5_ air pollution directly. The air pollution prevention BI model of parents from both groups forms paths models that vary in complexity. Our results clearly distinguish the different path models between the parents of both groups.

### Implications and further research

The development and understanding of preventative behaviors can be diverse. In this study AT was shown that it does not affect the BI of the parents from both groups. But other studies have shown that this is not the case. According to Gifford and Nilsson [51], cultural and ethnic differences can affect pro-environmental behaviors. Fang [2] pointed out that ‘There are often many different environmental concerns between different races and ethnic groups, and cognitive differences can emerge due to cultural differences” Fang [2] also suggested that these differences were always related to overall thought structures and the logic of different cultures. In the backgrounds of Hong Kong, the meaning of education is important, so do other Asian cultures. ‘Western to an Eastern culture where differences in cultural values and norms’ among education [52].

The TPB model has been criticized for not containing belief factors in the first version [53, 54]. Ajzen [1] added behavioral beliefs before AT, normative beliefs before SN, and control beliefs before PBC [42]. Therefore, additional studies on environmental behaviors related to air pollution can and should include dimensions that address behavioral, normative, and control beliefs. In addition, additional variables can be added across different countries for a deeper analysis.

## Supplementary Information

**Additional file 1.**

## Data Availability

All data generated or analysed during this study are included in this published article.

## Abbreviations

AT: Attitudes
SN: Subjective norms
PBC: Perceived behavioral controls
BI: Behavioral intentions
PLS-SEM: Partial least squares regression
