# Supplementary material for: Predicting the protective behavioral intentions for parents with young children that possess different levels of education in Hong Kong using the theory of planned behavior for air polluted with PM2.5
Source: BMC Public Health. 2022 Apr 15;22:761. doi: 10.1186/s12889-022-13141-9 (PMC9011999; doi:10.1186/s12889-022-13141-9)
Supplement: Supplementary file 1 — Additional file 1. [file 12889_2022_13141_MOESM1_ESM.docx]

**Questionnaire (English version)**

Five-point Likert scale (1=strongly disagree to 5=strongly agree)

**Predicting the Protective Behavioral Intentions for parents with young children that possess different levels of education in Hong Kong using the Theory of Planned Behavior for air polluted with PM_2.5_**

**1. Questions about Attitudes (AT)**

AT 1. I am worried about air pollution in Hong Kong.

AT 2. I care about recycling and utilization of daily life resources.

AT 3. I care about whether the air polluted with PM _2.5_ affects the health of myself and my family.

AT 4. I am concerned about whether the air polluted with PM _2.5_ affects the health of community residents.

AT 5. I care about whether the air polluted with PM _2.5_ affects the health of the citizens.

AT 6. I care about environmental issues arising from industry and manufacturing.

AT 7. I care about environmental issues arising from economic development.

**2. Questions about Subjective Norms (SN)**

SN 1. Most people who are important to me support me by not eating barbecued food.

SN 2. Most people who are important to me support me by walking, cycling or taking public transportation to go out.

SN 3. Most people who are important to me support me when I wear masks for myself and my children when air pollution occurs.

SN 4. Most people who are important to me support me when I participate in environmental protection activities to improve air pollution.

**3. Questions about Perceived Behavioral Controls (PBC)**

PBC 1. I can skip eating barbecued food to reduce air pollution.

PBC 2. I can walk, bike, or take public transportation to reduce air pollution.

PBC 3. Although wearing a mask is troublesome, I can remind my children to use a mask when the air polluted with PM _2.5_ is severe.

PBC 4. When the air polluted with PM _2.5_ is severe, I can guide children to wear a mask and protect their face.

**4. Questions about Behavioral Intentions (BI)**

BI 1. I don’t eat barbecued food to avoid air pollutants.

BI 2. I can walk and cycle or take public transportation to go out to reduce air pollution.

BI 3. Even if I spend more, I will use fuel with less environmental impact.

BI 4. I will advise others not to pollute the environment.

BI 5. When the air pollution is serious, I will let the child wear a mask and protect their face.

BI 6. When the Air Pollution Health Index (AQHI) reaches a "very high" level, I will let the child stay indoors (the second highest level among the five levels).

BI 7. I will pay attention to the Air Quality Health Index (AQHI) every day to remind children to pay attention to air pollution protection (AQHI).
